# Transcriptomic de novo analysis of pitaya (*Hylocereus polyrhizus*) canker disease caused by *Neoscytalidium dimidiatum*

**DOI:** 10.1186/s12864-018-5343-0

**Published:** 2019-01-07

**Authors:** Min Xu, Cheng-Li Liu, Juan Luo, Zhao Qi, Zhen Yan, Yu Fu, Shuang-Shuang Wei, Hua Tang

**Affiliations:** 1 Hainan Key Laboratory for Sustainable Utilization of Tropical Bioresources, Institute of Tropical Agriculture and Forestry, Hainan University, No.58 Renmin Avenue, Haikou, 570228 Hainan People’s Republic of China; 2 University of Sanya, No.191 Yingbin Avenue Xueyuan Road, Sanya, 572000 Hainan People’s Republic of China

**Keywords:** Red-fleshed pitaya, Transcriptomic, Canker disease, RNA-sequencing, Differentially expressed genes

## Abstract

**Background:**

Canker disease caused by *Neoscytalidium dimidiatum* is the most serious disease that attacks the pitaya industry. One pathogenic fungus, referred to as *ND8*, was isolated from the wild-type red-fleshed pitaya (*Hylocereus polyrhizus*) of Hainan Province. In the early stages of this disease, stems show little spots and a loss of green color. These spots then gradually spread until the stems became rotten due to infection by various strains. Canker disease caused by *Neoscytalidium dimidiatum* poses a significant threat to pitaya commercial plantations with the growth of stems and the yields, quality of pitaya fruits. However, a lack of transcriptomic and genomic information hinders our understanding of the molecular mechanisms underlying the pitaya defense response.

**Results:**

We investigated the host responses of red-fleshed pitaya (*H. polyrhizus*) cultivars against *N. dimidiatum* using Illumina RNA-Seq technology. Significant expression profiles of 23 defense-related genes were further analyzed by qRT-PCR. The total read length based on RNA-Seq was 25,010,007; mean length was 744, the N50 was 1206, and the guanine-cytosine content was 44.48%. Our investigation evaluated 33,584 unigenes, of which 6209 (18.49%) and 27,375 (81.51%) were contigs and singlets, respectively. These unigenes shared a similarity of 16.62% with *Vitis vinifera*, 7.48% with *Theobroma cacao*, 6.6% with *Nelumbo nucifera* and 5.35% with *Jatropha curcas*. The assembled unigenes were annotated into non-redundant (NR, 25161 unigenes), Kyoto Encyclopedia of Genes and Genomes (KEGG, 17895 unigenes), Clusters of Orthologous Groups (COG, 10475 unigenes), InterPro (19,045 unigenes), and Swiss-Prot public protein databases (16,458 unigenes). In addition, 24 differentially expressed genes, which were mainly associated with plant pathology pathways, were analyzed in-depth.

**Conclusions:**

This study provides a basis for further in-depth research on the protein function of the annotated unigene assembly with cDNA sequences.

## Background

Pitaya, also known as dragon fruit, is an important tropical and subtropical fruit tree. At present, there are two known species of pitaya: the red-fleshed pitaya (*Hylocereus polyrhizus*) and the white-fleshed pitaya (*Hylocereus undatus*). However, the most cultivated and commercialized species is the red-fleshed pitaya with its red pulp and pericarp (*H. polyrhizus*) that is enriched with anthocyanins [1, 2]. Pitaya belongs to the Cactaceae and its native tropical zone is Latin America, which includes Mexico, and Central and South America [3]. Currently, it is also extensively grown throughout tropical and subtropical regions worldwide [4], especially in Asian countries, such as Malaysia, Vietnam, Thailand, Philippines and China. Presently, the main areas of pitaya cultivation (especially the red dragon fruits) include the Hainan, Guangxi, Guangdong, Yunnan, Fujian and Taiwan provinces of China.

**Fig. 1:**
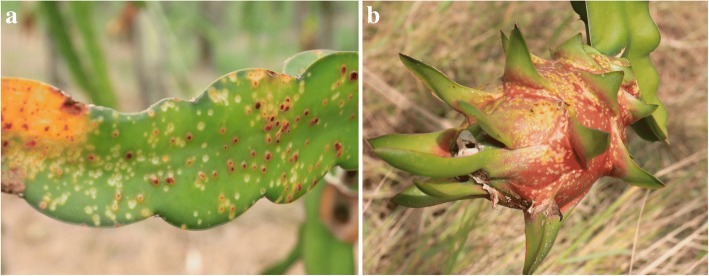
The impact of the canker disease on the pitaya stems and fruits. **a** Diseased stems in pitaya plantation; **b** Diseased fruits in pitaya plantation

Pitaya has been proven to be very adaptable to environmental conditions and disease pressures. However, the rapid spread of stem canker, a disease caused by *Neoscytalidium dimidiatum* among red-fleshed pitaya (*H. polyrhizus*), has become a serious threat to some commercial plantations (Fig. 1). The disease was first reported in Taiwan province of China, and features a distinctive canker on stems and fruits, and quickly spread to most commercial cultivation areas [5]. Shortly thereafter, it was discovered and reported in Guangdong Province of China, Malaysia, Israel, and Florida with similar characteristics [6–10]. Various studies have reported the incidence of canker disease caused by *Neoscytalidium dimidiatum* and have provided basic information relating to the identity and isolation of pathogenic strains.

Generally, two main recognition systems are associated with the response to an attack by the microorganism. One system recognizes the pathogen-associated molecular patterns (PAMPs) via pattern-recognition receptors (PRRs), which are localized on the membrane and activate an innate immune response, or more specifically PAMP-triggered immunity (PTI) [11]. The PAMPs are structural molecules, such as bacterial peptidoglycan and flagellin, oomycete glucans, lipopolysaccharide harpin, xylanase and chitin [12, 13]. In contrast, PRRs are cell surface-localized receptors, most of which are receptor-like kinases (RLKs) or receptor-like proteins (RLPs). The RLKs contain extracellular, transmembrane, and cytoplasmic kinase domains, whereas the RLPs lack the kinase domain [14]. A burst of reactive oxygen species (ROS) is a typical reaction in early cellular PTI events; activation of the mitogen-activated protein kinase (MAPK) cascade and expression of immune-related genes are also associated with such events [15]. The second system is mediated by intracellular immune receptors, which directly or indirectly recognize virulence effectors of pathogens that are secreted within host cells, thereby inducing effector-triggered immunity (ETI). In the second system, pathogen effectors are recognized by intracellular receptors, which are referred to as resistance (R) proteins, and mainly include leucine-rich repeats (LRR) or the LRR-like family, toll/interleukin-1-receptor (TIR), and serine/threonine kinases (S/TK) to activate the ETI response [14, 16]. Compared with PTI, the activation of ETI by receptors localized to the nucleus appears to be more directly associated with transcriptional regulation of the expression of defense genes [17]. However, some ambiguous theories exist regarding PTI and ETI, as it also appears that both can be robust or weak, depending on the interaction, defense signaling pathways, receptors and environmental conditions [13].

The RNA-Sequencing (RNA-Seq) technique is an excellent method that permits quick and extensive comprehension of the transcriptional level of genes in a variety of plant species using next-generation sequencing technologies [18]. Here, to investigate the genes associated with the defense response of pitaya to canker disease caused by *Neoscytalidium dimidiatum* in PTI and ETI systems, we used RNA-Seq technology to monitor the transcriptomic profiles of red-fleshed pitaya (*H. polyrhizus*) in response to fungal invasion. A comparison was made between normal and diseased stem tissuses during transcriptomic analysis, and the significant differentially expressed genes (DEGs) were analyzed in detail. These DEGs were annotated in public protein databases, suggesting that they may play important roles in PTI and ETI systems in response to the invasion of *Neoscytalidium dimidiatum*. The aim of the present study was to identify the key genes that encode steps in the plant pathological pathways of pitaya. This article provides a solid basis for a better understanding of the transcriptomic response of the red-fleshed pitaya (*H. polyrhizus*) to canker disease.

## Results

### Sequencing, de novo assembly and annotation

**Fig. 2:**
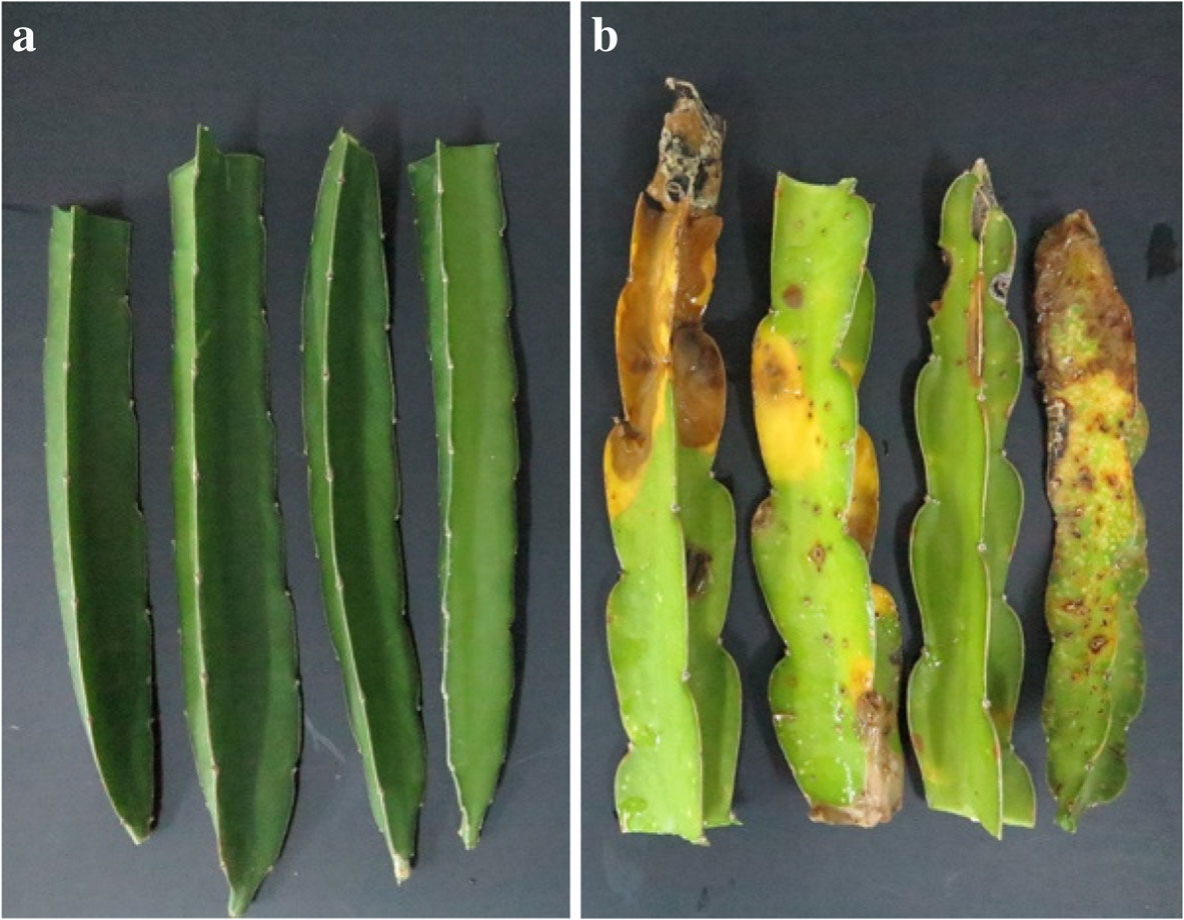
Pitaya tissues used for de novo transcriptomic analysis. **a** Normal stems; **b** Diseased stems

Healthy and diseased stem tissues of red-fleshed pitaya (*H. polyrhizus*) (Fig. 2) were collected from the orchard of Ledong County, Hainan Province. Three normal samples (N1, N2, and N3), and three diseased samples (D1, D2, and D3), were used for transcriptional sequencing. However, N1 and D2 examples were abandoned because the results were not consistent. The unigenes assembled from the four remaining samples were then merged. In total, we evaluated 33,584 unigenes, of which 6209 (18.49%) and 27,375 (81.51%) were contigs and singlets, respectively. The total read length was 25,010,007, mean length was 744 bp, the N50 was 1206, and the guanine-cytosine (GC) content was 44.48%, based on RNA-Seq. The range of the unigene lengths in the four samples was 497–702 bp. Details relating to the assembled information from each sample and the merged results are shown in Table 1. In total, 1608 DEGs, with a fold change ≥2.00 or a fold change ≤0.5, and a false discovery rate (FDR) ≤ 0.001, were monitored. On average, the number of unigenes from D samples was 45.7% higher than other samples, reflecting a significant increase in the number of sequences resulting from fungal infection.

**Table 1:** Results of assembled samples

Sample	Total number	Total length	Mean length	N50	GC%
N2	17227	9167524	532	686	45.07
N3	13331	6633532	497	610	45.43
D1	25583	16144615	631	920	45.12
D3	30702	21582053	702	1081	44.48
All-Unigene	33584	25010007	744	1206	44.66

GC: guanine-cytosine

**Fig. 3:**
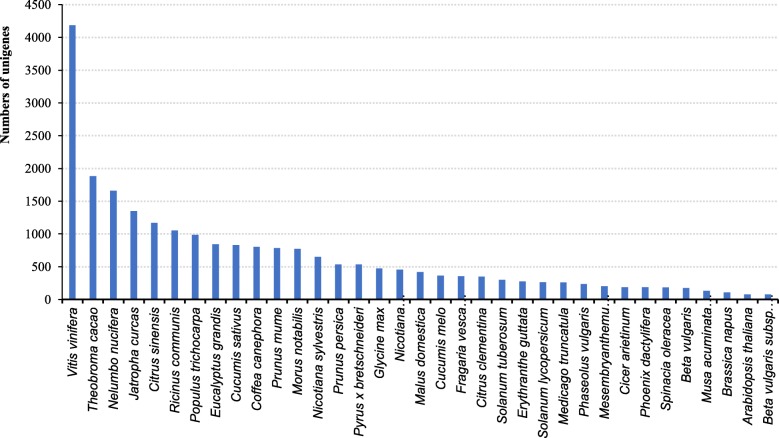
Species distribution of all unigenes. The x-axis shows the species of annotated unigenes while the y-axis shows the number of unigenes

**Fig. 4:**
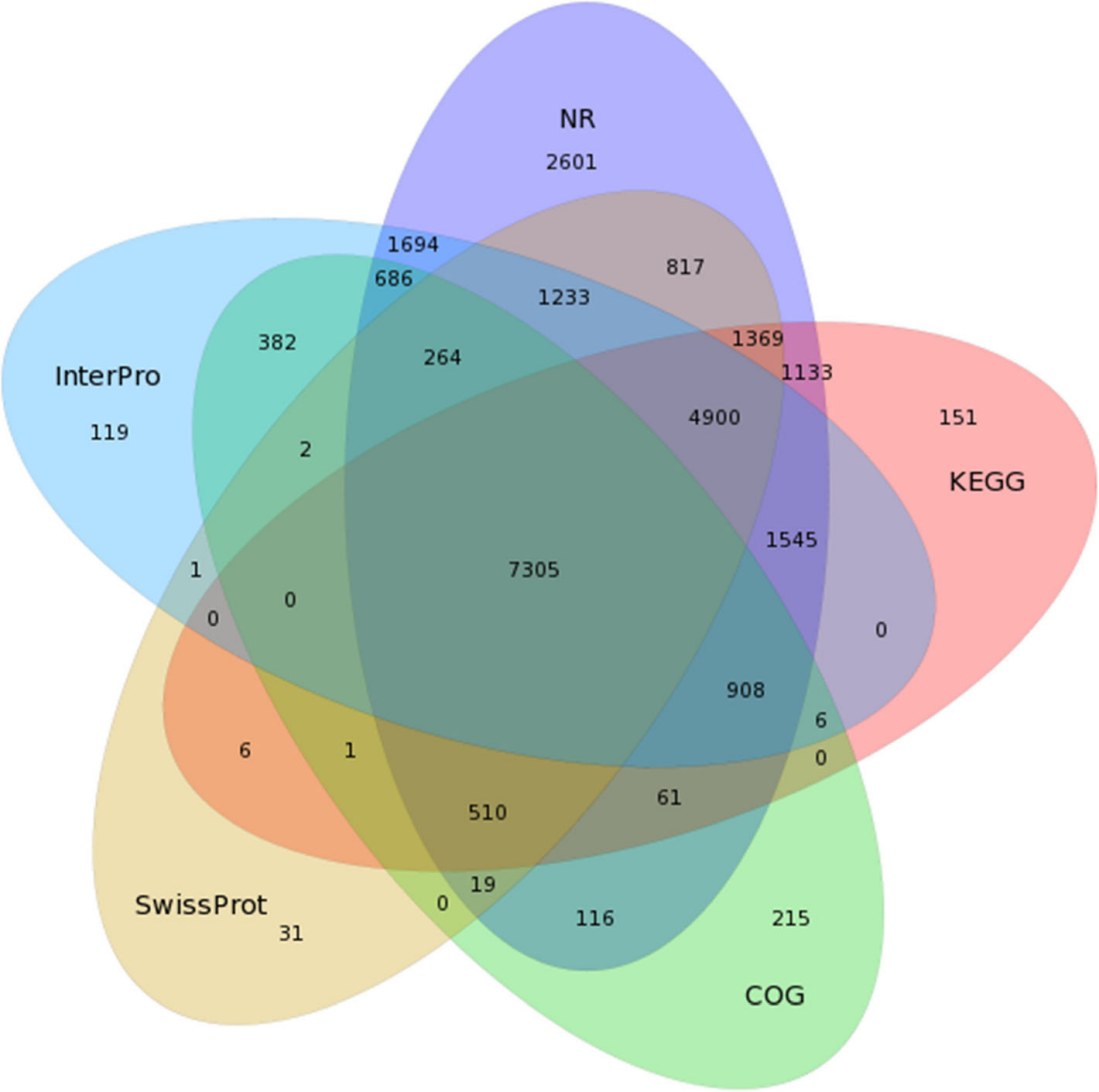
Venn diagram of unigenes annotated in NR, KEGG, COG, InterPro, and SwissProt protein databases

Owing to the lack of a reference genome for *H. polyrhizus*, the assembled unigenes were blast searched into the Non-redundant (NR), Kyoto Encyclopedia of Genes and Genomes (KEGG), Clusters of Orthologous Groups (COG), InterPro, and SwissProt public protein databases using search tools. The species with the greatest number of *H. polyrhizus* unigenes were *Vitis vinifera* (16.62%), *Theobroma cacao* (7.48%), *Nelumbo nucifera* (6.6%), and *Jatropha curcas* (5.35%), as shown in Fig. 3. Creation of a Venn diagram (Fig. 4), showed that there were 7305 unigenes annotated in all databases, 25,161 unigenes annotated in the NR database, 17,895 unigenes annotated in the KEGG database, 10,475 unigenes annotated in the COG database, 19,045 unigenes annotated in the InterPro database, and 16,458 Unigenes annotated in the SwissProt database. These findings provide a basis for further in-depth research on protein function of the annotated unigenes, which were assembled with cDNA sequences.

### Gene ontology (GO) functional annotation of DEGs

**Fig. 5:**
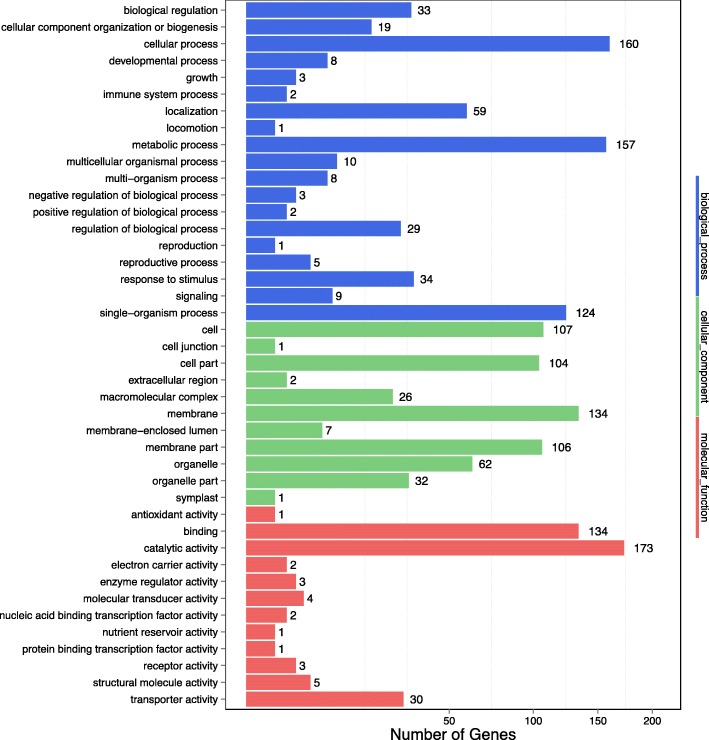
Gene Ontology (GO) annotation of differentially expressed genes (DEGs). The x-axis shows the number of genes while the y-axis shows gene function annotation of GO categories

Based on GO functional annotation, approximately 1608 DEGs were classified into three essential categories, namely biological process, cellular component, and molecular function. The unigenes we identified covered a wide range of functional GO categories. Of the 1608 DEGs classified, most were associated with catalytic activity (173 DEGs, 10.75%), binding (134 DEGs, 8.33%), and transporter (30 DEGs, 1.87%) aspects of molecular function. The biological processes were associated with cellular processes (160 DEGs, 9.95%), metabolic processes (157 DEGs, 9.76%), and single-organism processes (124 DEGs, 7.71%). The cellular component processes were mainly associated with the membrane (134 DEGs, 8.33%), cell (107 DEGs, 6.65%), membrane part (106 DEGs, 6.59%), and cell part (104 DEGs, 6.47%) aspects (Fig. 5). Analysis of the GO terms of pitaya transcripts showed that these genes might have been associated with kinases (catalytic activity) and receptors (cellular and metabolic processes, and the membrane).

### DEGs associated with plant-pathogen interactions

**Fig. 6:**
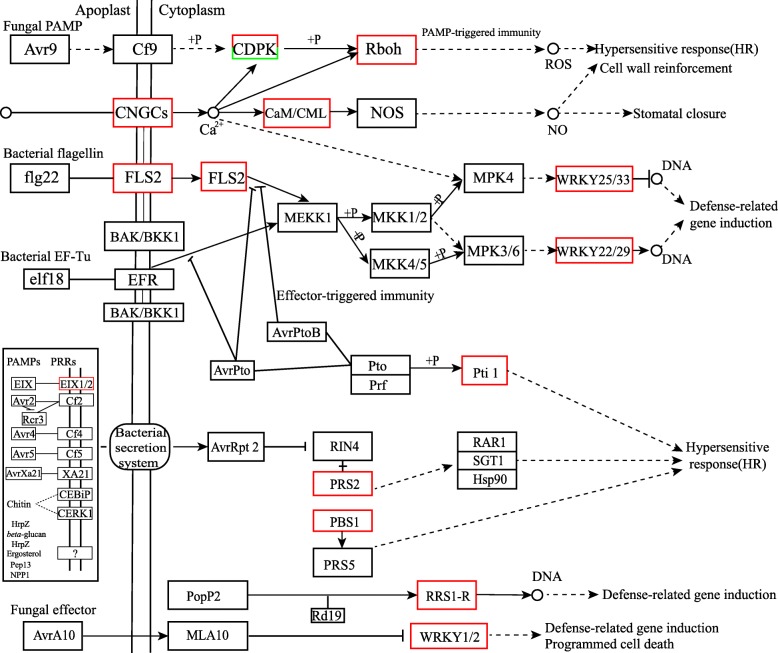
Differentially expressed genes (DEGs) associated with plant-pathogen interactions in *H. polyrhizus*. The original plant-pathogen interaction pathway was downloaded from the KEGG database (https://www.genome.jp/dbget-bin/www_bget?map04626). Genes in red frames were up-regulated, those in green frames were down-regulated, and those in black frames showed no change in regulation

Twenty-four DEGs were associated with plant-pathogen interaction pathways; of these, 23 were up-regulated and one was down-regulated. These unigenes play different roles in the plant-pathogen interaction pathways. Unigene13603_All and Unigene11517_All were annotated in the cyclic nucleotide-gated channel (CNGC) family, which mediates Ca^2+^ influx from cellular stores in plants [19]. The CNGCs are reportedly Ca^2+^-permeable channels that interact with the ubiquitous Ca^2+^ sensor calmodulin, and play important roles in the response to plant hormones, biotic and abiotic stresses, and plant immunity [20–22]. Unigene12122_All, Unigene16412_All, and CL2472.Contig2_All were annotated in the calcium-dependent protein kinase family (CDPK). The CDPKs are crucial sensors of changes in calcium concentration and play multiple roles in plant development and growth, abscisic acid (ABA)-mediated processes, jasmonic acid (JA) biosynthesis, plant tolerance to stress, and plant fungal stimuli interaction [23–28]. The CDPKs are phosphorylated by phosphatase and participate in the hypersensitive response (HR). Furthermore, another calcium-related gene, Unigene7838_All, was annotated in two types of Ca2^+^-sensing proteins, calmodulin and calmodulin-like (CaM/CML) (Fig. 6). The CaM/CML protein family plays complex and versatile roles in modulating cellular responses to various stimuli, particularly biotic stresses in grapevine [29]. The ROS produced by respiratory burst oxidase homologs are associated with multiple signal transduction pathways in diverse biological processes in plants [30]. In our research, an up-regulated Unigene (13565_All) was annotated in the respiratory burst oxidase homologs (Rboh) membrane-bound enzyme family, which is also known as the NADPH oxidases [31]. In tomato plants, Rboh activity and H_2_O_2_ signaling are important components of melatonin-induced stress tolerance [32]. This unigene may therefore encode the most critical enzyme of pitaya associated with ROS production under conditions of *Neoscytalidium dimidiatum* infection.

A plant-pathogen interaction pathway (Fig. 6) showed that the flagellin-sensing 2 (FLS2) family was significantly up-regulated, and included Unigene13867_All, Unigene9087_All, CL1260.Contig2_All, Unigene13234_All, Unigene24332_All, and Unigene15298_All. Furthermore, FLS2 is a well-known PRR kinase that recognizes a conserved 22 amino acid N-terminal sequence of the bacterial flagellin protein (flg22) [33]. Previous research has shown that FLS2 contains three domains, which include an extracellular LRR domain, a transmembrane domain and a cytoplasmic kinase domain [34]. The FLS2 receptor complex is directly coupled with *Arabidopsis* heterotrimeric G proteins to regulate immune signaling via both pre-activation and post-activation mechanisms [35]. Our transcriptome data showed that LRR receptor-like serine/threonine-protein kinases (LRR-RLKs) were differentially expressed and included Unigene13867_All, CL1043.Contig1_All, and Unigene9087_All. The LRR-RLKs play pivotal roles in plant growth, development, cellular signal transduction, brassinosteroid (BR) and ABA signal pathways, and responses to biotic and abiotic stresses [36]. The unigenes we detected that were associated with FLS2 may play important roles in the HR and defense-related gene induction in response to fungal infection.

**Fig. 7:**
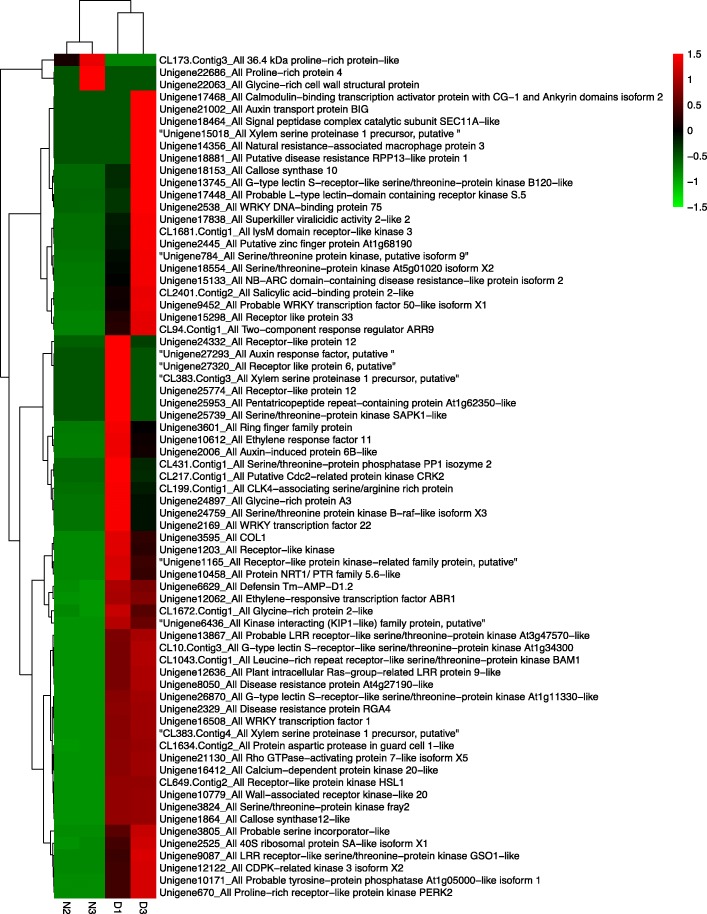
Heatmap of differentially expressed genes (DEGs) related to the defense response. Red indicates up-regulated expression, whereas green indicates down-regulated expression

Moreover, three LRR classes of plant disease resistance genes PRS [37] (Unigene8050_All, Unigene15133_All, and Unigene14674_All); a resistance *PBS* gene (Unigene24332_All) that encodes a serine/threonine-protein kinase [38]; a pto-interacting protein gene PTI (Unigene24688_All) that encodes a serine/threonine kinase [39]; and a receptor for the fungal elicitor ethylene-inducing xylanase (EIX) that binds with EIX to induce the HR in tobacco and tomato [40] (Unigene24332_All), were significantly up-regulated in pathogen interaction pathways. These DEGs may participate in the HR, defense-related gene induction or programmed cell death (Fig. 6). The expression profiles of DEGs in four samples asociated with plant-pathogen interactions are presented in the pheatmap show in Fig. 7, and in Table 2.

**Table 2:** Expression profiles of unigenes associated with plant pathological interactions

Unigene family	Unigene ID	Down/up regulation	KO number	N-Expression	D-Expression	Annotation
CDPK	Unigene12122_All	Up	K13412	0.01	1.565	CDPK-related kinase 3 isoform X2
	Unigene16412_All	Up	K13412	0.01	1.27	calcium-dependent protein kinase 20-like
	CL2472.Contig2_All	Down	K13412	919.92	7.415	unnamed protein product
Rboh	Unigene13565_All	Up	K13447	0.01	0.95	hypothetical protein CISIN_1g002711mg
CNGCs	Unigene13603_All	Up	K05391	0.01	1.35	putative potassium channel protein Kmt1p
	Unigene11517_All	Up	K05391	0.01	1.05	Cyclic nucleotide-binding transporter 1 isoform 8
CaM/CML	Unigene7838_All	Up	K13448	0.01	1.32	uncharacterized protein LOC100248601
FLS2	Unigene13867_All	Up	K13420	0.01	1.84	probable LRR receptor-like serine/threonine-protein kinase
	Unigene9087_All	Up	K13420	0.01	1.75	LRR receptor-like serine/threonine-protein kinase GSO1-like
	CL1260.Contig2_All	Up	K13420	0.01	1.64	hypothetical protein POPTR_0016s02970g
	Unigene13234_All	Up	K13420	0.01	1.485	uncharacterized protein LOC103419002
	Unigene24332_All	Up	K13420	0.01	1.065	Receptor-like protein 12
	Unigene15298_All	Up	K13420	0.01	0.955	Receptor-like protein 33
WRKY25/33	Unigene9452_All	Up	K13424	0.01	1.25	WRKY transcription factor 50-like isoform X1
WRKY22/29	Unigene2169_All	Up	K13425	0.01	1.355	WRKY transcription factor 22
EIX1/2	Unigene24332_All	Up	K13466	0.01	1.065	Receptor-like protein 12
RPS2	Unigene8050_All	Up	K13459	0.01	1.455	disease resistance protein At4g27190-like
	Unigene15133_All	Up	K13459	0.01	0.96	NB-ARC domain-containing disease resistance-like protein
PBS1	Unigene5397_All	Up	K13430	0.01	1.215	hypothetical protein JCGZ_18014
PTI 1	Unigene24688_All	Up	K13436	0.01	1.435	NADPH: quinone oxidoreductase-like
RRS1-R	Unigene14674_All	Up	K16225	0.01	1.395	unnamed protein product
WRKY1/2	Unigene14674_All	Up	K18835	0.01	1.395	unnamed protein product
	Unigene2538_All	Up	K18835	0.065	3.63	WRKY DNA-binding protein 75
	Unigene9452_All	Up	K18834	0.01	1.25	WRKY transcription factor 50-like isoform X1
	Unigene16508_All	Up	K18834	0.01	1.065	WRKY transcription factor 1

### Predicted expression of transcription factors (TFs)

**Fig. 8:**
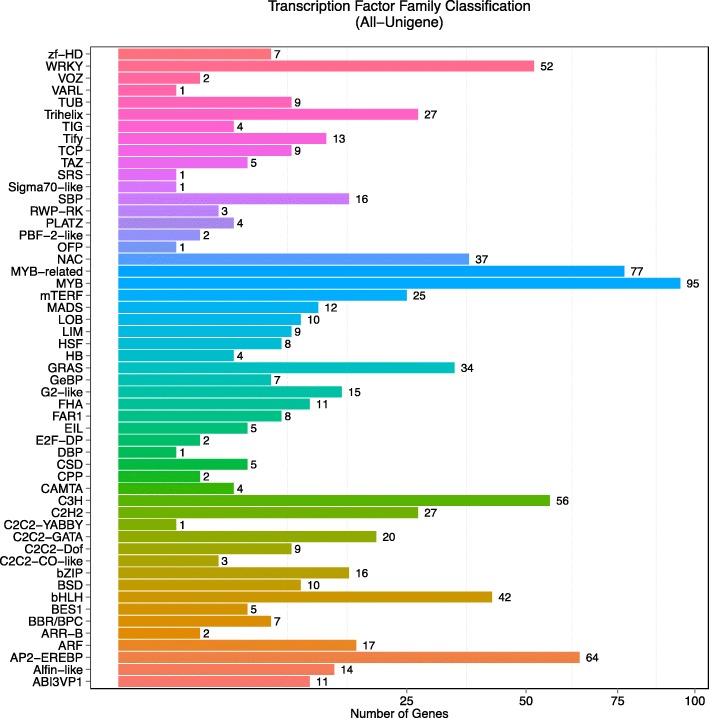
Differentially expressed genes (DEGs) and transcription factor (TF) family classification. The x-axis shows the number of genes while the y-axis shows TF family classification

Transcription factors (TFs) are proteins that, when in contact with RNA polymerase, can confirm a transcription initiation complex and participate in the transcription initiation process. Generally, a TF contains a transcriptional activation/repression domain and a transcriptional binding domain [41]. As a regulator, a TF regulates the expression of genes downstream of a pathway by binding with DNA sequences either by itself or by interacting with other TFs or proteins. TFs are associated with the significant mechanisms of the regulation of disease resistance genes and receptors for cellular localization and activation, as well as DNA binding and transcription activities [42]. In the merged data of all samples in the present study, a total of 832 transcription factors were predicted, and the majority of the TF families were categorized as *MYB* (95, 11.42%); MYB-related (77, 9.25%); APETALA2/ethylene-responsive element binding protein (AP2-EREBP, 64, 7.69%); C3H (56, 6.73%); WRKY (52, 6.25%); bHLH (42, 5.05%); NAC (37, 4.45%); and GRAS (34, 4.09%), among others (Fig. 8).

The TFs make essential contributions to the processes of growth, development, flowering, and biotic and abiotic stresses in the plant. For instance, the family of MYB factors include the conserved MYB DNA-binding domain and appears to be involved in the regulation of the cell cycle in animals, plants and other higher eukaryotes [43]. The *AP2-EREBP* genes contained the AP2 family, RAV (related to ABI3/MP) family, and ERF (ethylene response factor) family, based on the number of AP2 domains and sequence similarity [44]. The *AP2-EREBP* gene reportedly plays roles in controlling flower and seed developmental processes, regulatory networks in response to hormones, pathogen attacks and environmental signals [45, 46]. Moreover, NAC TFs reportedly also take part in the plant-pathogen interactions of *Arabidopsis*, potato, maize, and wheat in response to wounding or pathogen attack as a transcriptional regulator [47]. In our plant pathology interaction pathways, WRKYs (Unigene9452_All, Unigene2169_All, Unigene14674_All, Unigene2538_All, and Unigene16508_All) were significantly and differentially expressed. The WRKY proteins can interact with themselves, VQ-domain proteins, chromatin remodeling proteins, calmodulin-like proteins or some essential MAP kinases [48]. The unigenes identified in the present study may act as downstream products of the MAPK pathway to activate the expression of defense-related genes, or recognize the fungal effector, which may result in the induction of defense-related genes or programmed cell death (Fig. 6). The differentially expressed TFs under investigation may play essential roles in growth, development, leaf senescence and responses to biotic and abiotic stimuli.

### qRT-PCR verification

**Fig. 9:**
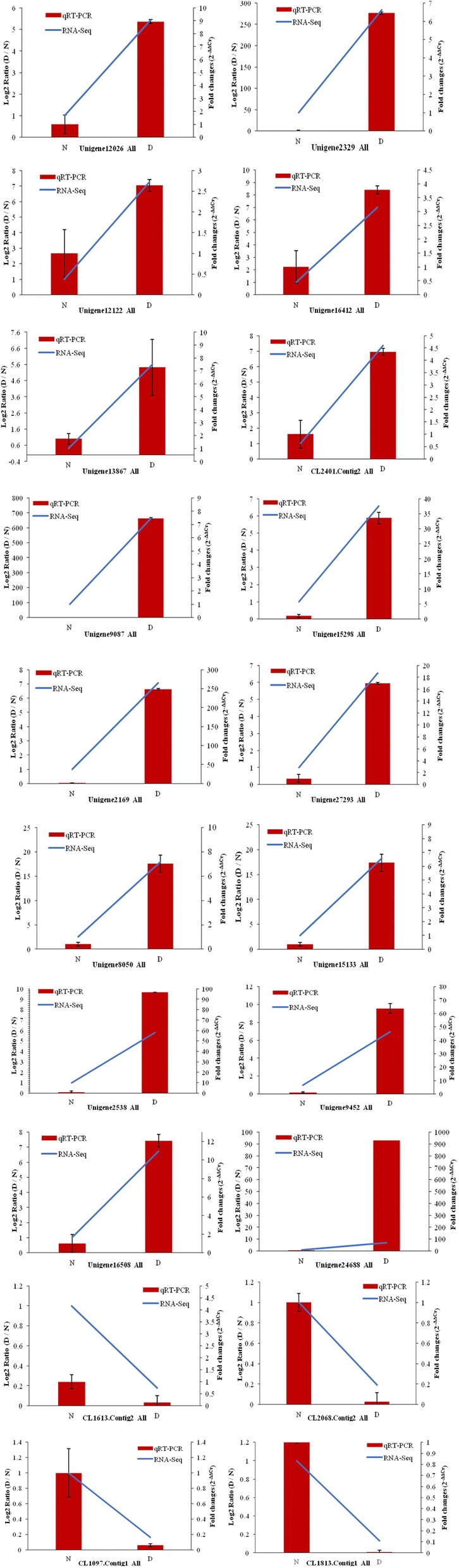
Validation of gene expression by quantitative real-time polymerase chain reaction (qRT-PCR). Gene expression levels were measured by qRT-PCR and compared with RNA-Seq results. Histograms represent the fold changes of genes (D3/N3) by qRT-PCR, whereas line charts represent gene expression according to the log2 ratio (fragments per kilobase of transcript per million mapped reads (FPKM) of D/FPKM of N) in RNA-Seq. All genes selected for qRT-PCR analysis were analyzed in three biological replicates. Bars represent the ± SEΔCт of three experiments. Details of the genes selected for qRT-PCR are provided in Table 3

To validate the RNA-Seq data, 23 unigenes (Table 3) that were significantly expressed, most of which were associated with phytopathology, were selected for additional qRT-PCR analysis. These unigenes included one ethylene-responsive TF (Unigene12062_All); two LRR receptor-like serine/threonine-protein kinases (Unigene13867_All and Unigene9087_All); one salicylic acid-binding protein (CL2401.Contig2_All); two CDPK-related kinases (Unigene12122_All and Unigene16412_All); three disease resistance proteins (Unigene2329_All, Unigene8050_All, and Unigene15133_All); two RLPs (Unigene24332_All and Unigene15298_All); four WRKY TFs (Unigene9452_All, Unigene2169_All, Unigene2538_All, and Unigene16508_All); one auxin response factor (Unigene27293_All); one NADPH-related protein (Unigene24688_All); one lipoxygenase protein (CL1613.Contig2_All); and five differentially expressed proteins (CL2068.Contig2_All, CL1097.Contig1_All, CL1813.Contig1_All, Unigene2331_All, and Unigene9233_All). These genes were either significantly up- or down-regulated in diseased tissues. The results of the qRT-PCR assay showed that 20 of the 23 genes selected for qRT-PCR (three biological replicates) conformed to the RNA-Seq data, with an accuracy of 87%. These results (Fig. 9) indicated that the RNA-Seq findings were both stable and reliable.

**Table 3:** Gene expression in normal (N) and diseased (D) tissues based on qRT-PCR

Unigene	N-Expression	D-Expression	Log2 fold change (D/N)	Up/down regulation	Annotation
Unigene12062_All	0.175	7.605	5.442	Up	Ethylene - responsive transcription factor ABR1
Unigene13867_All	0.01	1.84	7.524	Up	probable LRR receptor-like serine / threonine-protein kinase At3g47570 - like
CL2401.Contig2_All	0.01	1.65	7.366	Up	Salicylic acid-binding protein 2-like
Unigene12122_All	0.01	1.565	7.290	Up	CDPK-related kinase 3 isoform X2
Unigene16412_All	0.01	1.27	6.989	Up	Calcium-dependent protein kinase 20-like
Unigene2329_All	0.01	0.98	6.615	Up	Disease resistance protein RGA4
Unigene9087_All	0.01	1.75	7.451	Up	LRR receptor-like serine/threonine-protein Kinase GSO1-like
Unigene24332_All	0.01	1.065	6.735	Up	Receptor-like protein 12
Unigene15298_All	0.01	0.955	6.577	Up	Receptor-like protein 33
Unigene9452_All	0.01	1.25	6.966	Up	probable WRKY transcription factor 50-like isoform X1
Unigene2169_All	0.01	1.355	7.082	Up	WRKY transcription factor 22
Unigene27293_All	0.01	0.945	6.562	Up	Auxin response factor, putative
Unigene8050_All	0.01	1.455	7.185	Up	Disease resistance protein At4g27190-like
Unigene15133_All	0.01	0.96	6.585	Up	NB-ARC domain-containing disease resistance-like protein isoform 2
Unigene2538_All	0.065	3.63	5.803	Up	WRKY DNA-binding protein 75
Unigene16508_All	0.01	1.065	6.735	Up	WRKY transcription factor 1
Unigene24688_All	0.01	1.435	7.165	Up	NADPH: quinone oxidoreductase-like
CL1613.Contig2_All	17.795	0.41	-5.440	Down	Lipoxygenase homology domain - containing protein 1 - like
CL2068.Contig2_All	11.83	0.315	-5.231	Down	Organ-specific protein S2-like
CL1097.Contig1_All	11.045	0.16	-6.109	Down	Hypothetical protein POPTR_0001s31970g
CL1813.Contig1_All	2.49	0.01	-7.960	Down	Pathogenesis-related protein Bet v I family
Unigene2331_All	0.95	0.01	-6.570	Down	Beta - glucosidase 46 isoform X3
Unigene9233_All	1.21	0.01	-6.919	Down	Pherophorin-dz1 protein

## Discussion

The cultivation of pitaya is an emerging industry with a bright future and high commercial value, especially for red-fleshed pitaya (*H. polyrhizus*). However, canker disease caused by *Neoscytalidium dimidiatum* presents the most devastating attacks on pitaya plantations at present [9]. The most efficient solution to prevent this disease is to cut off the infected tissues and bury them in soil away from the plantation. In addition, molecular breeding is the best way to acquire disease-resistant cultivars; however, this is not easily done with pitaya. Moreover, additional tasks need to be completed to verify which cultivar is the most resistant among a variety of pitaya species.

Plant immunity is a complicated process that is effected mainly in response to extracellular or intracellular receptors that recognize PAMPs or effectors in PTI and ETI systems [13, 14]. The defense response mechanisms and roles of the receptors have been previously reported in *Arabidopsis*, tomato, and rice, among others [34, 35, 37, 39, 40, 49, 50], but has been scarcely described in pitaya. In our transcriptome results, higher expression levels of unigenes were detected in *H. polyrhizus* and 24 DEGs associated with the plant-pathogen interaction pathway. Most of these genes are signal receptors, protein kinases, and TFs, which may play critical roles in the plant-pathogen interaction networks.

Calcium acts as an important conserved second messenger in plant immune and stress responses [51]. Calcium is sensed by Ca^2+^-binding proteins in signaling networks, including calmodulin (CaM), calmodulin-like (CML), CDPKs, and calcineurin B-like proteins [19, 52]. The CNGCs are calmodulin-permeable cation transport channels that have the potential to integrate signals from cyclic nucleotide and Ca^2+^ signaling pathways [53]. In our transcriptome analysis, seven unigenes (Table 2) related to Ca^2+^ levels and signaling pathways were annotated and showed significant changes in expression levels in diseased red-fleshed pitaya. These genes included two CNGCs related genes (Unigene13603_All and Unigene11517_All); one CaM/CML (Unigene7838_All); three CDPK genes (Unigene12122_All, Unigene16412_All, and CL2472.Contig2); and one Rboh family gene (Unigene13565_All). These genes act as receptors or kinases in the HR, cell wall reinforcement, and stomatal closure (Fig. 6).

Pathogenesis-related (PR) proteins comprise one of the major sources of plant-derived allergens, and are generally induced by various types of pathogens, such as viruses, bacteria and fungi [54]. In pepper, PR4b reportedly interacts with LRR proteins and PR4c, as a plasma membrane-localized cysteine protease inhibitor, to trigger cell death and the defense signaling response [55, 56]. In the present study, we also monitored four differentially expressed PR homologous proteins, which were annotated in the SwissProt and InterPro public protein databases. Among these four proteins, two were up-regulated (Unigene9313_All and Unigene11745_All), and the other two were down-regulated (CL1813.Contig1_All and CL1271.Contig2_All). Further work, involving protein function verification and genetic transformation, is now being carried out to investigate their roles in the response to the fungal infection of *H. polyrhizus*.

**Fig. 10:**
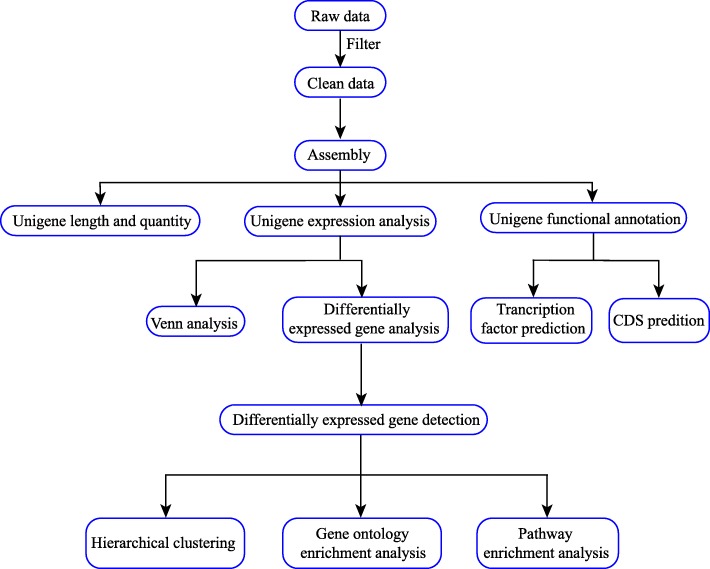
Bioinformatics analysis of the de novo transcriptome study processes

A total of 832 TFs were monitored in the present study. The TF proteins are key factors in the regulatory networks that control development, metabolism, and the responses to biotic and abiotic stresses in numerous plant species. In our RNA-Seq data, *MYB* and MYB-related family genes comprised the largest group, which accounted for 20.67%. A MYB domain protein, COLORED1 (C1), which is associated with anthocyanin synthesis, was first identified in maize [57]. The MYB TF family is a major player in drought and cold responses [58, 59], the phenylpropanoid metabolic pathway [60], and JA and ABA signal transduction pathways [61, 62]. The NAC TFs comprise a large protein family, with 138 putative NAC genes in *Arabidopsis*, as listed in the Plant Transcription Factor Database (PlantTFDB, http://planttfdb.cbi.pku.edu.cn/) [63]. However, 37 (4.45%) putative NAC genes in *H. polyrhizus* were identified based on our RNA-Seq data*.* The NAC family of genes have a highly conserved N-terminal DNA-binding domain and a variable C-terminal domain. The NAC TFs participate in the response to drought, salinity, and/or low temperatures and regulate JA-signaled defense responses in various plants [64, 65]. In addition, 52 WRKY superfamily TF genes were monitored in the present study and five unigenes (Unigene 9452_All, Unigene 14674_All, Unigene 2538_All, Unigene 2169_All, and Unigene 16508_All) were differentially expressed upstream in red pitaya. The WRKY genes are quite common in plants, which contain a conserved N-terminal sequence of WRKYGQK, along with a Zn finger-like motif (C2H2 type or C2HC type) [66]. The trend in qRT-PCR expression levels of four WRKY unigenes (Unigene 9452_All, Unigene 2169_All, Unigene 16508_All, and Unigene 2538_All) were consistent with the RNA-Seq data (Fig. 10 and Table 3). The Unigene 14674_All was an unnamed protein product, which was annotated to both *WRKY* and *RRS1-R* gene families. Pathway results suggested that the five differentially expressed WRKY unigenes were important candidate genes of *H. polyrhizus* involved in plant pathology interactions (Fig. 6).

## Conclusions

Our findings may pave a fundamental step to a better understanding of the defense responses of pitaya. We have provided hypotheses for the functions of the genes identified, according to public protein annotation databases. However, specific details of the roles of these genes in pitaya defense responses pose a relevant and interesting challenge. Thus, further research is now required to clarify how significant DEGs regulate pitaya defense processes via genetic transformation, when infected by *Neoscytalidium dimidiatum*.

At present, transgenic research is the best method with which to clarify the functions of a gene. However, this is difficult to achieve in tropical and subtropical non-model plants. Three major difficulties are associated with transgenic research in pitaya. Firstly, there have been problems associated with the establishment of a tissue culture system. Secondly, we need to consider the stability of the tissue culture system and the resolution of issues if stability is unfavorable. Finally, the period required to screen a disease-resistant cultivar is relatively long. Nevertheless, in spite of the difficulties faced in the study of non-model plants, transgenic Cavendish bananas with resistance to Fusarium wilt tropical race 4 were reported recently following a 3-year field trial [67]. That was a major breakthrough for the banana industry and could also imply a brighter future for the pitaya industry. Thus, the problems associated with transgenic pitaya should be solved eventually with further broad-scale research.

## Methods

### Plant materials and culture conditions

Healthy and diseased stem tissues of red-fleshed pitaya (*H. polyrhizus*) were collected from the orchard of Ledong County, Hainan Province. These plants had been growing for approximately five years in a plantation under natural conditions. Fungal spores were isolated from diseased tissues, and morphological and molecular analyses were conducted to determine the species of *Neoscytalidium dimidiatum* 8 (*ND8*). The article about isolation of *ND8* has been published in Australasian Plant Pathology [68]. The collected stem tissues were divided into two groups with similar characteristics in triplicate within each group. The stems were cut into pieces and immediately frozen in liquid nitrogen. The three replicates of the normal group were named N1, N2, and N3, whereas the three replicates of the diseased group were named D1, D2, and D3. Three biological replicates, each consisting of four stem cutting bases, were frozen in liquid nitrogen and stored at − 80 °C until further analysis. Sample tissues were transferred to Shenzhen BGI Tech Company (Shenzhen, China) for transcriptome de novo assembly and sequencing using an Illumina HiSeq system.

### Total RNA extraction

Tissues (100 mg) were placed into a precooled mortar to be ground into a powder with liquid nitrogen. The ground stem powder was collected into a precooled 2 mL microcentrifuge tube containing 2 mL RNase-free cetyltrimethylammonium bromide (CTAB) extracting solution, which contained 0.1% diethyl pyrocarbonate (DEPC), 2.5% CTAB, 1.4 M NaCl, 20 mM EDTA, 100 mM Tris-HCl (pH 8.0), and 4% polyvinylpyrrolidone (PVP). The tubes were then placed in a water bath at 65 °C for 30 min to induce cell lysis, and centrifuged at 13,000 rpm for 5 min at 25–28 °C. The residue was discarded, and the supernatant was transferred into a clean 2 mL microcentrifuge tube. Trichloromethane (1 mL) was added to the supernatant solution, mixed lightly, and centrifuged at 13000 rpm for 15 min at 4 °C. The supernatant was then transferred into a clean 2 mL microcentrifuge tube, and this procedure was repeated once more. Subsequently, 8 M lithium chloride was added with RNase-free water to 0.6 volume of the supernatant and left overnight. The mixture was centrifuged for 30 min at 13000 rpm and 4 °C to obtain an RNA precipitate. RNase-free ethyl alcohol (75%) was used to wash the RNA precipitate twice. Finally, RNA was dissolved in RNase-free ddH_2_O and stored at − 80 °C until further use.

### Construction of the cDNA library and de novo RNA-Seq

Poly (A) mRNA from the total RNA was enriched by poly-T oligo-attached magnetic beads (Invitrogen, Thermo Fisher Scientific, CN), according to the manufacturer’s protocol. Purified mRNA was then divided into short fragments by mixing with fragmentation buffer. First strand cDNA was synthesized using a random hexamer primer. Buffer, dNTPs, RNase H, and DNA polymerase I were then added to the fragmented mRNA templates to synthesize second-strand cDNA. The double-stranded cDNA was purified using magnetic beads. End repair, and the addition of 3′-end single nucleotide A (adenine), were then performed. Finally, sequencing adaptors were ligated to the fragments, which were enriched by PCR amplification. During the quality control step, a 2100 Bioanalyzer (Agilent, Santa Clara, CA, USA) and a StepOnePlus Real-Time PCR System (Applied Biosystems Inc., Foster City, CA, USA) were used to qualify and quantify sequences for the sample libraries. The constructed library products were finally prepared for sequencing using a HiSeq 2000 system (Illumina, San Diego, CA, USA) at the Beijing Genomics Institute (BGI).

### Filtering of raw reads and the assembly of clean reads

Before any further analysis could be conducted, we needed to consider quality control of the data. In addition, low-quality sequences were filtered out of the raw data to reduce data noise. We defined “dirty” raw reads as those that contained adapter sequence, high levels of unknown bases, and low-quality reads. These needed to be removed before data analysis. Filtering steps were as follows: 1) Reads with adapters were removed; 2) reads in which unknown bases were more than 10% were removed; and 3) low-quality reads were removed (the percentage of low-quality bases is usually over 50% in a read, and we defined a low-quality base to be one with a sequencing quality of no more than 5). After filtering, the remaining reads were referred to as “clean reads” and used for downstream bioinformatics analysis. Clean data were obtained and stored in the FASTQ format after filtering.

Clean reads were assembled using the Trinity paired-end assembly method [69]. The assembled clean reads, also referred to as transcripts, were clustered and this eliminated any redundancy in obtaining unigenes using the TIGR Gene Indices clustering tools (TGICL) [70]. For the study of multi-samples, the previous step using the TGICL for downstream analysis of the unigenes was repeated. The processed unigenes were referred to as “All-Unigenes.” All-Unigenes were divided into two parts; one part included clusters of a few unigenes with more than 70 similarities among them (starting with “CL,” followed by the gene family ID); and the other included singletons (starting with “Unigene”). The clean data arising from high-throughput sequencing (RNA-Seq) results, and the processed files, have been submitted to the Gene Expression Omnibus (GEO) database at the GenBank website with accession number GSE119976. The process of preliminary bioinformatics analysis was performed according to the flow diagram in Fig. 10.

### Functional annotation and the detection of differentially expressed unigenes

After assembly, *H. polyrhizus* pitaya All unigenes (contigs and singletons) were functionally aligned to the public protein databases, NR, GO, KEGG, COG, InterPro, and SwissProt. All unigenes were annotated according to their sequence similarity to previously annotated genes in these public databases. The phyper function of R software (https://www.r-project.org) was used for enrichment analysis, according to the results of GO and KEGG pathway gene annotations in functional analyses. The *p*-value was corrected by the FDR. We considered that unigenes with a FDR ≤ 0.01 were significantly expressed in the GO and KEGG pathways. Moreover, NOIseq [71] and PassionDis [72] methods were used to test DEGs. A fold change ≥2.00 or fold change ≤0.5, with a Probability ≥0.8 in NOIseq, and fold change ≥2.00 or fold change ≤0.5, with a FDR ≤ 0.001 in PassionDis of unigenes were used to define DEGs in the normal and diseased libraries. According to the results of the DEGs test, the pheatmap function of R software was used for hierarchical clustering analysis.

### Verification of DEGs by quantitative reverse transcription PCR (qRT-PCR)

To investigate the stability and accuracy of RNA-Seq, 23 DEGs that were possibly associated with plant pathology, were selected for real-time quantitative RT-PCR (qRT-PCR). Total RNA extraction was performed as described in previous steps. Following treatment with DNase I (Thermo Fisher Scientific, USA), first strand cDNA synthesis was conducted according to the manufacturer’s protocols using the PrimeScript™ 1st strand cDNA synthesis kit (code No.6210A, Takara, Japan). qRT-PCR was performed using a 20-μL reaction system, containing 10 μL of SYBR® Premix Ex TaqII (Tli RNase Plus) (2×), 2 μL cDNA templates, 0.8 μL PCR forward primer (10 μM), 0.8 μL PCR reverse primer (10 μM), 0.4 μL ROX reference Dye II (50×) and 6 μL of nuclease-free water. The qRT-PCR assays (three technical replicates) were carried out using a 7500 Applied Biosystems qRT-PCR System (Life Tech, USA). The primers of all genes selected for qRT-PCR were designed using Primer Premier 6 software (www.premierbiosoft.com). The pitaya ubiquitin gene (*UBQ*) was used as an internal reference control for data normalization. The primers for *UBQ* were as follows: F: 5′-TGAATCATCCGACACCA-3′; and R: 5′-TCCTCTTCTTAGCACCACC-3′ [73]. Reactions were carried out under the following conditions: 50 °C for 2 min, 95 °C for 10 min, followed by 40 cycles at 95 °C for 15 s, and 60 °C for 1 min. Output data were generated using the Applied Biosystems 7500 software version 2.0.6 (ABI, America). All reactions were performed with two independent biological replicates, and the expression levels calculated for each sample were based on three technical replicates. The 2^-ΔΔCT^ method was used for qRT-PCR data analysis, in which PC_T. (test)_ = C_T (target, test)_ - C_T (reference, check)_; ΔC_T (calibrator)_ = C_T (target, calibrator)_ - C_T (reference, calibrator);_ and ΔΔC_T_ = ΔC_T (test)_ - ΔC_T (calibrator)_. The expression ratio of the experimental and control groups was 2^-ΔΔCT^.

## Abbreviations

ABA: abscisic acid
BR: brassinosteroid
CaM/CML: calmodulin and calmodulin-like
CDPK: calcium-dependent protein kinase
CNGC: cyclic nucleotide-gated channel
COG: Clusters of Orthologous Groups
DEGs: differentially expressed genes
EIX: ethylene-inducing xylanase
ERF: ethylene response factor
ETI: effector-triggered immunity
FDR: false discovery rate
FLS2: flagellin-sensing 2
GC: guanine-cytosine
HR: hypersensitive response
JA: jasmonic acid
KEGG: Kyoto Encyclopedia of Genes and Genomes
LRR: leucine-rich repeats
LRR-RLKs: LRR receptor-like serine/threonine-protein kinases
MAPK: mitogen-activated protein kinase
NR: Non-redundant
PAMPs: pathogen-associated molecular patterns
PR: Pathogenesis-related
PRRs: pattern-recognition receptors
PTI: PAMP-triggered immunity
Rboh: respiratory burst oxidase homologs
RLKs: receptor-like kinases
RLPs: receptor-like proteins
RNA-Seq: RNA-Sequencing
ROS: Reactive oxygen species
S/TK: serine/threonine kinases
TF: Transcription factor
TIR: toll/interleukin-1-receptor
